# Nutritional Status of the Cauliflower Cultivar ‘Verona’ Grown with Omission of out Added Macronutrients

**DOI:** 10.1371/journal.pone.0123500

**Published:** 2015-04-09

**Authors:** Matheus Saraiva Bianco, Arthur Bernardes Cecílio Filho, Leonardo Bianco de Carvalho

**Affiliations:** 1 Plant Production Department, Faculdade de Ciências Agrárias e Veterinárias, Universidade Estadual Paulista, Jaboticabal, São Paulo, Brasil; 2 Department of Agronomy, Centro de Ciências Agroveterinárias, Universidade do Estado de Santa Catarina, Lages, Santa Catarina, Brasil; University of Sassari, ITALY

## Abstract

Knowledge of plant nutritional status allows an understanding of the physiological responses of plants to crop fertilization. A hydroponic experiment evaluated the symptoms of macronutrient deficiency in cauliflower ‘Verona’ and determined: a) the macronutrient contents of foliar tissues when visual symptoms were observed, b) macronutrients content of foliar and inflorescence tissues at harvest. The effect of nutrient deficiency on inflorescence mass was also evaluated. Nitrogen deficiency caused chlorosis followed by purple color in the old leaves, while P deficiency caused only chlorosis in old leaves. Chlorosis at the edge of old leaves progressing to the center of the leaves was observed with the omission of K, and after was observed necrosis in the chlorotic areas. Ca deficiency caused tip burn in new leaves, while Mg deficiency caused internerval chlorosis in old leaves. The omission of each macronutrient reduced inflorescence dry matter. This deleterious effect was larger for N, P, and K deficiencies, reducing inflorescence dry matter by 87, 49, and 42%, respectively. When the nutrient solutions without N, P, K, Ca, or Mg were supplied to cauliflower plants, the macronutrient contents at harvest were 8.8, 0.6, 3.5, 13.0, and 0.8 g kg^-1^ in the foliar tissues and 27.3, 2.2, 21.6, 1.1, and 0.7 g kg^-1^ in the inflorescence tissues, respectively.

## Introduction

Nutritional status influences plant growth and development. Crop production and the quality of the harvested food are consequently affected. Knowledge of the nutritional status of plants allows physiological diagnosis and an interpretation of the ways by which plants respond to management conditions, especially crop fertilization.

Plant nutritional status can be diagnosed in various ways, but the most common method is to analyze the nutrient content of foliar tissues [1]. This method, however, is often not efficient due to the need for caution when collecting plant materials for analysis (leaf age, stage of plant growth, and leaf position) and the experimental determination of nutrient contents. Also, nutrient contents related to nutritional disorders have been poorly studied. The visual diagnosis of plant nutrition, though, has always aroused the interest of researchers, technicians, and growers worldwide, because it has no cost, is faster than laboratory methods, is good for use under field conditions [2, 3], and can be a very powerful diagnostic tool for evaluating the nutrient status of plants [2].

Avalhães et al. [4] observed yellowing in old cauliflower leaves when N was not supplied. Plant growth was slow when Phosphorus was not supplied, and older leaves were more intensely green, changing to purple in the central nervure. The omission of Potassium caused chlorosis in the board of the young leaves, and the central nervures were more intensely green. Plants cultivated without added Calcium had indented and warped leaves with necrotized tips. The omission of Magnesium caused plant wilting and chlorosis among the nervures of old leaves and necrosis of the board of the leaves. The petioles elongated followed by a decrease in leaf area when Sulfur was not supplied.

The evolution of the symptoms, from onset to the intermediate and final conditions, however, has not been photographed. Also, foliar nutrient contents at the time of viewing a deficiency have rarely been reported. The symptoms of nutrient deficiency are similar for many plants, but the expression of the symptoms varies due to the large diversity of plants and their environments [2].

The objectives of this study were: (a) to photograph and describe the visual symptoms of macronutrient deficiency in cauliflower, (b) to determine the macronutrient contents of the foliar tissues when the visual symptoms were observed, (c) to determine the macronutrient contents in the foliar and inflorescence tissues at harvest, and (d) to evaluate the effect of nutrient deficiency on inflorescence mass.

## Materials and Methods

### Plant material and growing conditions

An experiment under greenhouse conditions was conducted from January to May 2010 in Jaboticabal, São Paulo, Brazil (21°15′22′′S, 48°18′58′′W; 575 m a.s.l.). Hydroponic systems were used for supplying nutrients and water to plants of the cauliflower cultivar ‘Verona’. Cauliflower seeds were first scattered into small plates of phenolic foam containing 216 cells 2.5 cm in width, 2.5 cm in length, and 3.8 cm in height. Ten days after sowing, when the cotyledon leaves had fully expanded, the seedlings were transferred to an NFT (nutrient film technique) hydroponic system with polyethylene channels 5 cm in diameter and 6 cm apart. The seedlings remained in the NFT system for 24 days when they had four fully expanded leaves. The seedlings were then transplanted to polyvinyl chloride (PVC) hydroponic channels 20 cm in diameter and 200 cm in length covered with Tetra Pak paper. The PVC channels were maintained at 5% declivity and each channel linked to a 40-L nutrient-solution tank. Submersible aquarium pumps (Power Head CX-300D, Chosen Champion, USA) pumped the nutrient solutions in each tank at 1000 L h^-1^. The pumps operated from 7:00 a.m. to 6:00 p.m. without interruption and then for 15 min to 11:00 p.m., being switched on/off by a time controller.

### Nutrient solution and supply

Nutrient solutions for cauliflower cultivation have not been described, so we used a complete nutrient solution recommended for lettuce and other leafy vegetables [5]. A complete nutrient solution had concentrations of N-NH_4_^+^, N-NO_3_^-^, P, K, Ca, Mg, S, B, Cu, Fe, Mn, Mo, and Zn in of 19.2, 139.2, 31.2, 144.0, 114.0, 32.0, 41.6, 0.25, 0.02, 1.8, 0.4, 0.08, and 0.11 mg dm^-3^, respectively. A complete nutrient solution containing 80% macronutrients and 100% micronutrients was supplied to the seedlings from their transfer to the NFT system to 15 days after being transplanted to the PVC channels. The omission of macronutrients began when the plants had 12 fully expanded leaves. The pH of the nutrient solutions was adjusted with sulfuric acid when necessary. The nutrient solutions in each tank were completely replaced after 20 days.

### Treatments and experimental design

The treatments consisted of supplying six different nutrient solutions for cauliflower growth: a complete solution (CS) and identical solutions but without N (-N), without P (-P), without K (-K), without Ca (-Ca), and without Mg (-Mg). The treatments were arranged in a completely randomized design with four replicates. Each experimental plot consisted of a cultivation channel with four plants.

### Measurements

The time (in days) of the initial appearance of deficiency symptoms for each macronutrient was registered. The symptoms were photographed and described from their initial appearance to inflorescence harvesting, when the experiment ended. The number of leaves, leaf area, and foliar macronutrient contents at three distinct stages (young, intermediate, and old) were determined when the deficiency symptoms first appeared and at inflorescence harvesting. Leaf area was measured using a leaf-area meter (L-3100, Li-Cor, USA). The macronutrient contents of a standard leaf (a recently developed leaf) [6] were also determined for plants supplied with a complete nutrient solution when the deficiency symptoms of the macronutrients were first observed. The macronutrient contents of the inflorescences were also determined at harvest. The macronutrients were extracted following the protocols used by Bianco et al. [7] and Carvalho et al. [8]. Plant materials were washed, dried at 60–70°C in a forced-air convection oven for at least 96 hours, powdered using a Willey mill grinder with a 20-mesh steel screen, and stored in glass pots with silicon lids. The content of macronutrients was determined [9].

### Statistical analysis

The macronutrient contents were submitted to analyses of variance and Tukey’s test at 5% probability using SAS statistical software.

## Results and Discussion

**Fig 1:**
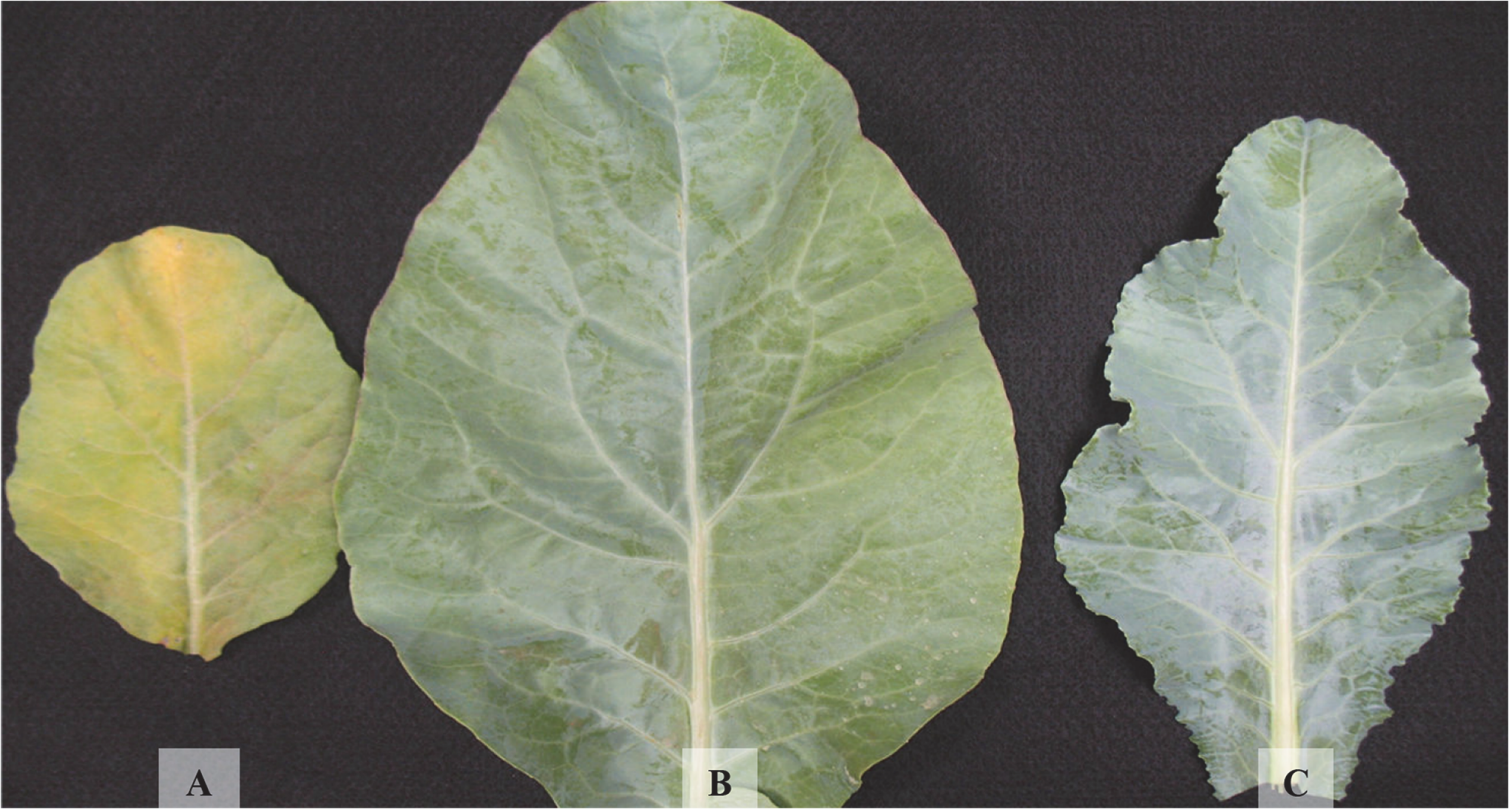
Old (A), intermediate (B), and young (C) leaves of the cauliflower ‘Verona’ 15 days after being supplied with a nutrient solution without N.

**Fig 2:**
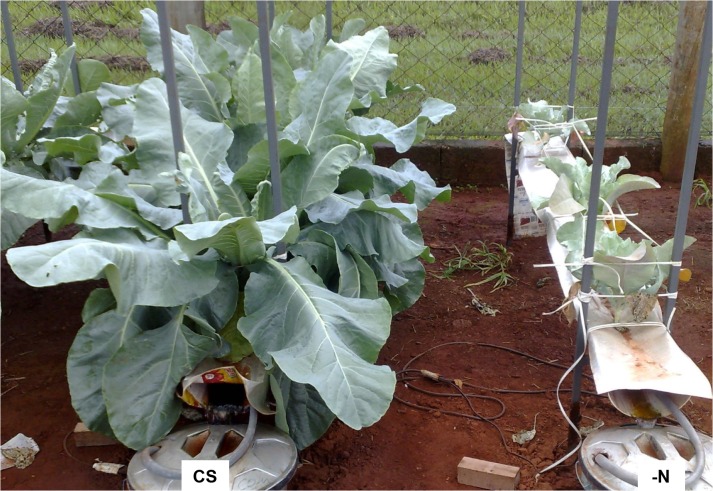
Plants of the cauliflower ‘Verona’ 45 days after being supplied with a complete nutrient solution (CS) and a nutrient solution without N (-N).

The initial symptoms of N deficiency were observed 15 days after the plants were supplied with a nutrient solution without N. The plants were 63 days old and had 14 leaves and a leaf area of 15.28 dm^2^. Old leaves became a lighter shade of green and subsequently yellowed (Fig 1), corroborating the findings by Chaterjee et al. [10]. By 30 days, the intermediate leaves also became light green, and the light-green old leaves became red and purple on the abaxial surface and yellow on the adaxial surface (Fig 2). These symptoms intensified over the growing cycle. Purpling on the abaxial surface of old leaves has also been observed in cabbage [11]. In cauliflower and other species of the family Brassicaceae, purple, red, or orange pigmentation commonly develops in leaves due to the degradation of chlorophyll molecules [10] and to the accumulation of anthocyanin molecules synthesized from carbohydrates not used for generating amino acids or other nitrogenous compounds [12]. The yellowing of old leaves has also been reported by Avalhães et al. [4], but not purpling.

When the deficiency symptoms were first observed (when the plants were 63 days old), the plants needed a large amount of N. ‘Verona’ had a large increase in the number of leaves and stem diameter and length between 30 and 75 days after sowing [13]. The lack of added N obviously had a very strong effect on plant growth, which affected crop productivity. The growth of the cauliflower plants without added N slowed from five days after the initial deficiency symptoms were observed, relative to the plants fertilized with a complete nutrient solution. A reduction in the number and size of leaves and in the dry mass of cauliflower plants has previously been reported under similar growing conditions [4]. Increasing doses of N, though, provided a significant increase in the number of leaves, stem diameter, and accumulation of macronutrients in *Brassica chinensis* var. *parachinensis* [14].

The reduction in plant growth was so intense that the plants growing under N deficiency for 45 days were smaller than well-fed plants (Fig 2). Growth reduction is one the main effects caused by N deficiency in plants [4, 12, 15]. N is the element that plants need most, because it is a constituent of amino acids, amides, proteins, nucleic acids, nucleotides, and coenzymes, so that N deficiency rapidly inhibits plant growth [12].

By 15 days after the plants were supplied with a nutrient solution without N, when the initial deficiency symptoms were observed, the N content of old leaves was 8.8 g kg^-1^, compared to 44.2 g kg^-1^ in the old leaves of plants supplied with a complete nutrient solution (Table 1 and S1 Table). The N contents of intermediate and young leaves were also lower, but no deficiency symptoms were observed. The contents of the other nutrients were also lower. The N content was 44.2 g kg^-1^ in a standard leaf of plants supplied with a complete nutrient solution, within an adequate range of 40–60 g kg^-1^ for well-fed plants [6]. Below an N content of 40 g kg^-1^, plants are considered to be deficient in N, as observed by Avalhães et al. [4] (11 g kg^-1^) and also in our study (8.8 g kg^-1^). The N contents of the shoots of cauliflower plants supplied with N have been reported as 41.2 g kg^-1^ [9], 36.3 g kg^-1^ [4], and 38.3 g kg^-1^ [16], and the foliar N content as between 40 and 21 g kg^-1^ between 20 and 70 days after transplanting, respectively [17]. Low N contents in old leaves can cause the proteolysis of proteins and the redistribution of amino acids, resulting in a collapse of chloroplasts and a decrease in chlorophyll content [18].

**Table 1:** Content of N, P, and K in leaves of cauliflower supplied with omission of some macronutrient.

NS	N			P			K		
	OL	IL	YL	OL	IL	YL	OL	IL	YL
**First Collection** ^**1**^									
**CS**	44.2 a	44.2 a	44.2 b	6.3 a	6.3 a	6.3 a	27.9 ab	27.9 a	27.9 a
**-N**	8.8 d	12.8 d	27.7 c	2.6 bc	2.7 bc	3.7 c	10.5 c	10.8 c	17.7 c
**-P**	20.9 cd	27.9 c	30.7 c	0.5 c	0.5 c	1.1 d	23.4 ab	17.8 b	18.4 c
**-K**	29.6 bc	33.5 bc	42.1 b	4.0 ab	4.1 ab	5.4 b	6.3 d	6.5 d	8.6 d
**-Ca**	37.1 ab	44.1 a	58.4 a	5.9 a	5.6 ab	5.9 ab	33.5 a	26.2 a	31.6 a
**-Mg**	29.6 bc	30.8 bc	44.1 b	4.4 ab	4.8 ab	5.2 b	17.8 bc	16.4 b	18.6 c
**Second Collection** ^**2**^									
**CS**	29.5 a	39.6 a	40.0 ab	6.1 a	6.1 a	6.4 a	33.9 b	29.9 ab	29.7 a
**-N**	6.0 b	7.8 c	12.6 d	1.5 c	1.7 d	2.3 d	6.7 d	11.7 c	18.3 d
**-P**	33.8 a	30.7 b	27.1 c	0.5 d	0.5 e	0.8 e	21.0 c	17.4 bc	19.2 cd
**-K**	28.7 a	31.9 b	45.9 a	6.0 a	5.8 ab	5.5 b	1.8 e	2.7 d	6.0 e
**-Ca**	28.4 a	32.6 b	34.0 bc	5.4 ab	4.4 c	4.5 c	43.1 a	33.5 ab	25.4 bc
**-Mg**	33.3 a	43.5 a	47.8 a	4.6 b	4.8 bc	5.9 ab	40.1 a	41.4 a	33.8 a

N, P, and K contents (g kg^-1^) of old (OL), intermediate (IL), and young (YL) leaves of the cauliflower ‘Verona’ supplied with a complete nutrient solution (CS) or a nutrient solution without added macronutrients (-N,-P,-K,-Ca, and-Mg).

^1^ The first collection was performed when deficiency symptoms first appeared, 15, 19, and 28 days after being supplied with nutrient solutions without N, P, or K, respectively.

^2^ The second collection was performed at inflorescence harvest. Means followed by the same letter in the same column did not differ significantly by Tukey’s test at 5% probability.

At harvest, the N contents in old, intermediate, and young leaves were 6.0, 7.8, and 12.6 g kg^-1^, respectively (Table 1 and S1 Table). The N content of inflorescences was lower in plants not supplied with N (27.3 g kg^-1^) than in plants supplied with a complete nutrient solution (41.8 g kg^-1^) (Table 2 and S4 Table). The N deficiency also reduced K, Ca, and Mg contents in the inflorescences. Alves et al. [17] observed an N content of 28 g kg^-1^ in ‘Verona’ inflorescences, and Camargo et al. [19] reported a high cauliflower production when inflorescences had 40 g kg^-1^ of N. Kano et al. [20] observed that the N content of inflorescences varied from 33.8 to 44.8 g kg^-1^, and N content increased linearly as more N was supplied to the plants. Both the leaves and inflorescences of cauliflower plants have higher levels of N than of other nutrients [13, 21, 22].

**Table 2:** Content of macronutrients in inflorescence of cauliflower supplied with omission of some macronutrient.

NS	N	P	K	Ca	Mg
**CS**	41.8 b	5.9 a	38.2 a	4.8 a	2.1 b
**-N**	27.3 c	5.0 a	32.9 b	3.0 b	2.2 b
**-P**	29.8 c	2.2 b	31.4 b	2.0 b	1.6 b
**-K**	71.7 a	6.4 a	21.6 c	4.1 a	2.5 a
**-Ca**	55.6 b	6.4 a	40.0 a	1.1 c	3.6 a
**-Mg**	44.8 b	6.3 a	35.9 a	4.8 a	0.7 c

Macronutrient contents (g kg^-1^) of inflorescences of the cauliflower ‘Verona’ supplied with a complete nutrient solution (CS) or a nutrient solution without added macronutrients (-N,-P,-K,-Ca, and-Mg). Means followed by the same letter in the same column did not differ significantly by Tukey’s test at 5% probability.

**Fig 3:**
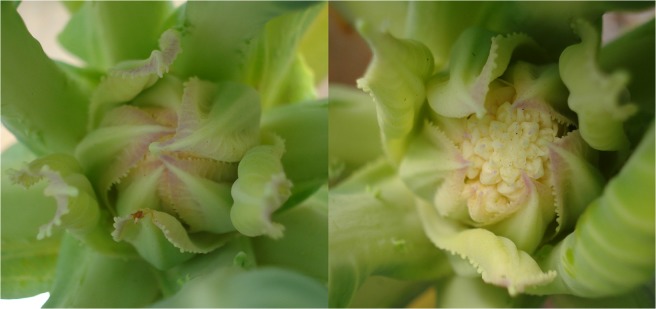
Detail of the layer of small green leaves covering the cauliflower ‘Verona’ head and the top of floral buds 92 days after being supplied with a nutrient solution without N.

N deficiency reduced the dry mass of inflorescences by 87% (130 g) relative to the inflorescences of plants supplied with a complete nutrient solution (1020 g), and the layer of small green leaves covering the cauliflower heads and the tops of the floral buds became red (Fig 3). The size and quality of the heads are the main characteristics affecting cauliflower marketing and are influenced by nutrients such as N, P, and B [19, 20, 23, 24]. Takeishi et al. [13] reported that 62% of the N was in the inflorescences of cauliflowers at harvest. Fresh mass, yield, and inflorescence N content of cauliflower plants increased linearly with the amount of N supplied (100–250 kg ha^-1^) [19, 20].

**Fig 4:**
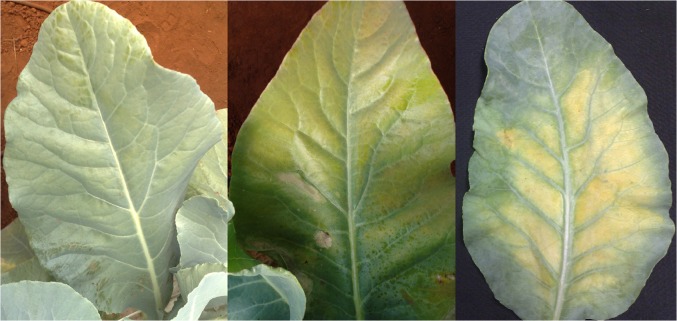
Progressive color changes in old leaves of the cauliflower ‘Verona’ supplied with a nutrient solution without P. Asymptomatic (A), beginning of yellowing (B), and advanced yellowing (C).

**Fig 5:**
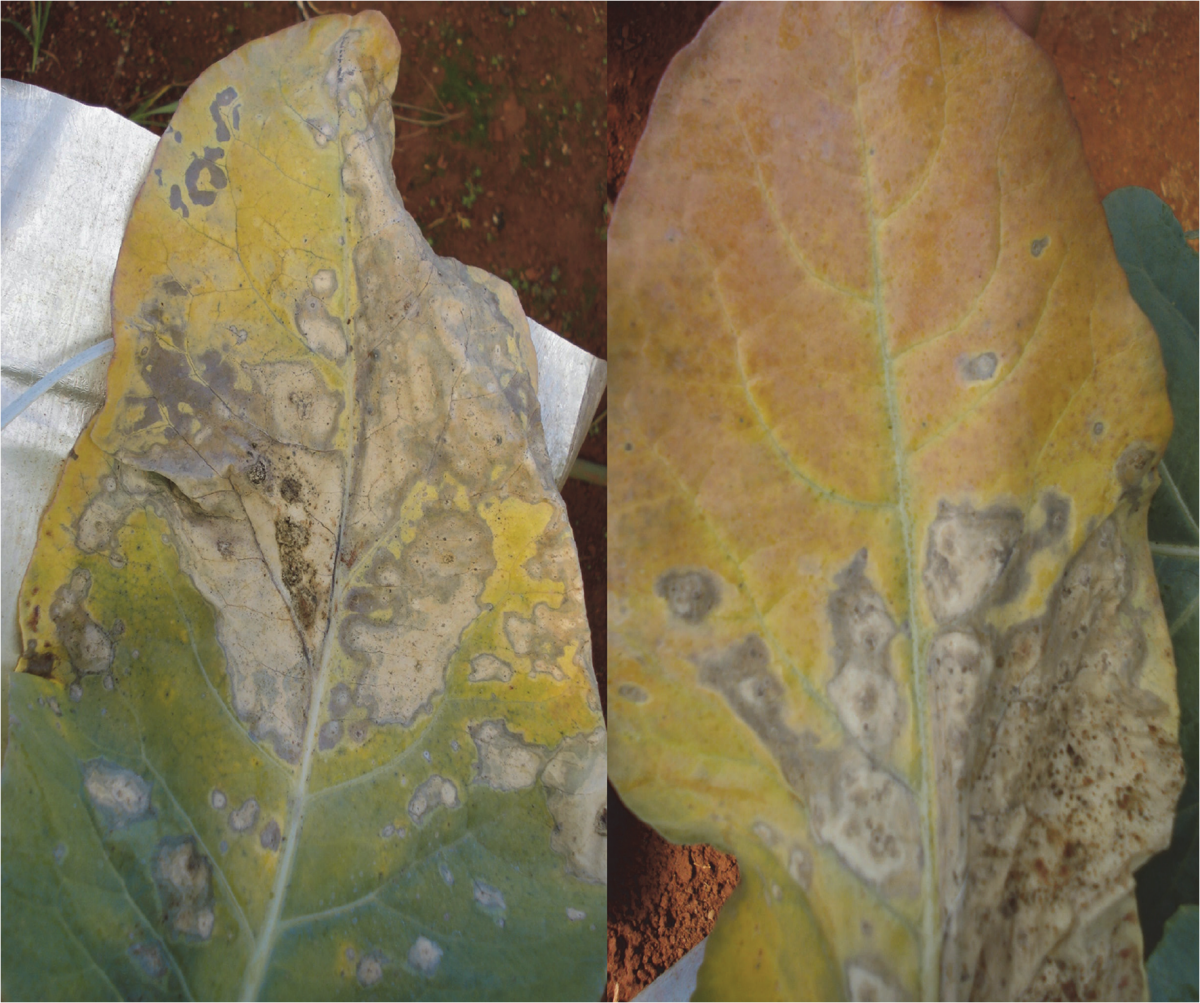
Old leaves of the cauliflower ‘Verona’, showing advanced symptoms of P deficiency, 40 days after being supplied with a nutrient solution without P.

**Fig 6:**
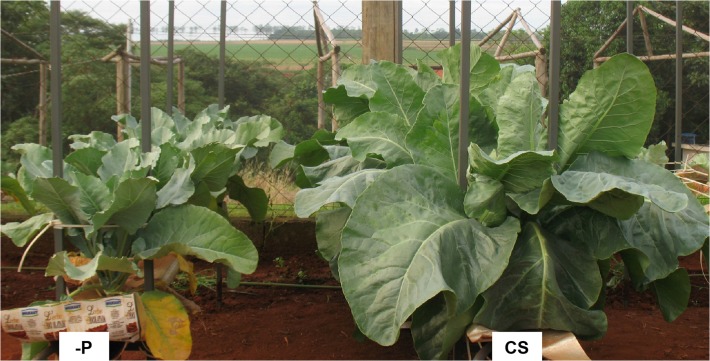
Plants of the cauliflower ‘Verona’ 45 days after being supplied with a complete nutrient solution (CS) and a nutrient solution without P (-P).

The initial symptoms of P deficiency were observed 19 days after the plants were supplied with a nutrient solution without P. The plants were 67 days old and had 15 leaves and a leaf area of 40.97 dm^2^. The shade of green progressively changed in old leaves, whose leaf blades eventually became a homogeneous yellow on both the adaxial and abaxial surfaces (Fig 4). Avalhães et al. [4], however, reported that the leaf blades of old leaves became dark green, and their nervures showed purpling. Dark-green younger leaves and purple or red old leaves are typical symptoms of P deficiency in plants [10]. In our study, previously yellowed old leaves had become necrotic 40 days after a nutrient solution without P was supplied (Fig 5), corroborating the findings by Bergmann [25]. Long-term P deficiency can lead to necrotic foliar tissues, especially at the leaf board, fallout, and the death of old leaves [25]. Plant growth decreased (Fig 6) because P is necessary for all physiological processes involving plant growth. Avalhães et al. [4] reported decreases in plant height, number of leaves, leaf area, and stem diameter of cauliflowers cultivated without P. Lynch et al. [26] also reported a decrease in the leaf area of cauliflower plants as a consequence of limiting P availability, due mainly to a reduction in the emergence and expansion of leaves.

The yellowing of old leaves may be due to the redistribution of P from old to young leaves for maintaining the metabolic activity, especially enzymatic activity, of young leaves [18], thereby drastically reducing the P content of old leaves and leading to chlorophyll degradation. Conroy et al. [27] observed structural changes of chloroplast thylakoid membranes, which reduced. plants to capture photons and, as a consequence, their use by photosystem II. The dysfunction of the photosynthetic apparatus could be the initial cause of some events leading to chlorophyll inefficiency and degradation in old leaves. A low photosynthetic efficiency per unit of chlorophyll is observed in plants with P deficiency [28]. Bottrill et al. [29] reported that P deficiency had the most deleterious effects on photosynthesis in spinach plants compared to other nutrient deficiencies. Severe P deficiency, producing low P contents in foliar tissues as observed in this study (Table 1 and S2 Table), probably limits anthocyanin synthesis. Red old leaves, as observed by Chatterjee et al. [10], were thus not observed in our study, likely due to a lack of anthocyanin synthesis.

Although only small amounts of P accumulates in cauliflower tissues [13, 21, 22], this plant has a high demand for P; only asparagus, tomato, and strawberry have higher demands [6]. In addition, more than 90% of the soil analyses in ‘Cerrado’ conditions of Brazil have indicated low concentrations of available P, and P availability decreases as the concentrations of clay and sesquioxides of iron and aluminum increase [18]. These factors are important for the improvement of P deficiency in cauliflower plants.

Nineteen days after the plants were supplied a nutrient solution without P, when the initial deficiency symptoms were observed, the P contents of old, intermediate, and young leaves were 0.5, 0.5, and 1.1 g kg^-1^, respectively (Table 1 and S2 Table). The P content was 6.3 g kg^-1^ in a standard leaf of plants supplied with a complete nutrient solution, within an adequate range of 4–7 g kg^-1^ for well-fed plants [6]. Avalhães et al. [4] found P contents of 1 and 5 g kg^-1^ in the foliar tissues of P-deficient and well-fed plants, respectively. P-deficient plants also had significant reductions in the other nutrient contents (Table 1 and S2 Table).

**Fig 7:**
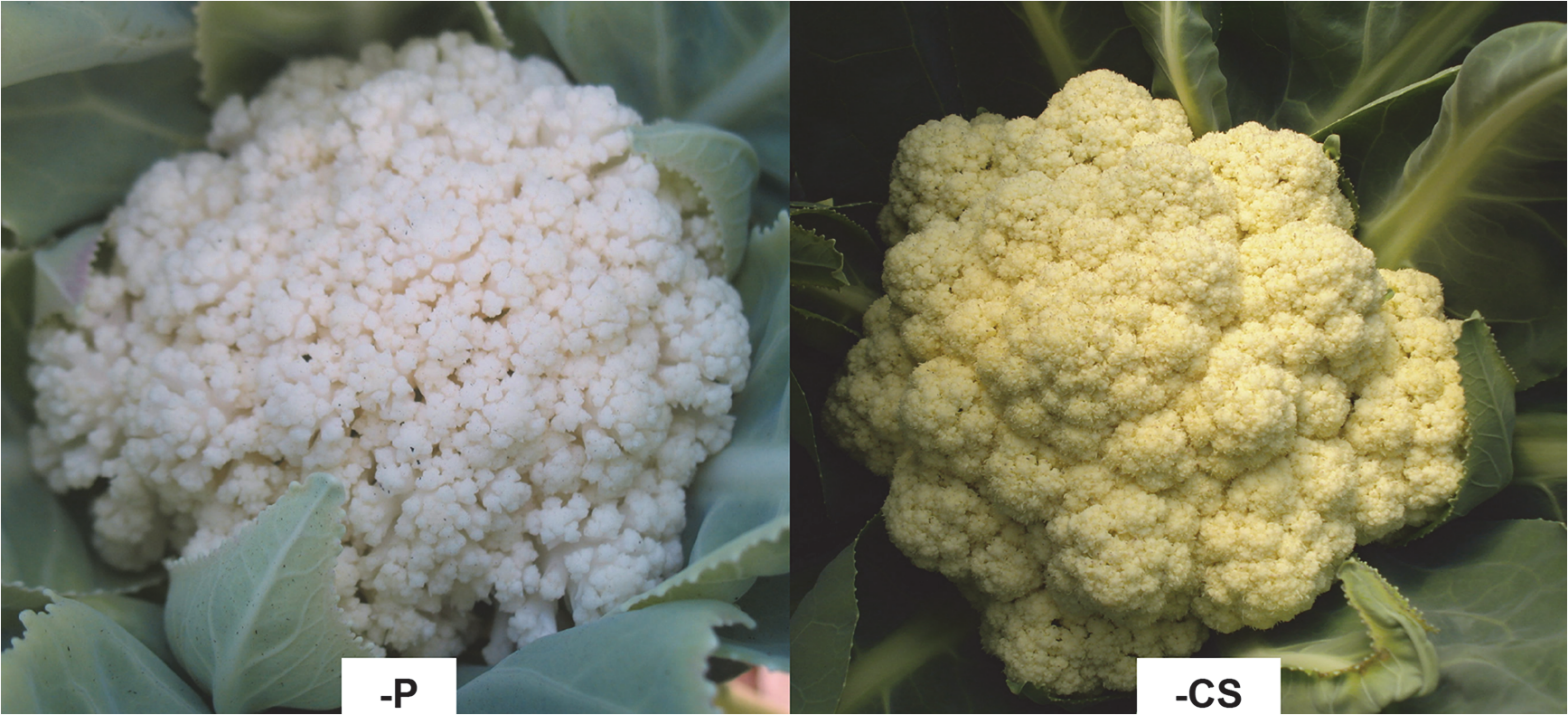
Detail of the cauliflower ‘Verona’ head at harvest supplied with a nutrient solution without P (-P) and a complete nutrient solution (CS).

The P contents at harvest of old, intermediate, and young leaves were 0.5, 0.5, and 0.8 g kg^-1^, respectively (Table 1 and S2 Table). The P contents of inflorescences were also lower in plants grown without added P (2.2 g kg^-1^) than in those supplied with a complete nutrient solution (5.9 g kg^-1^) (Table 2 and S4 Table). P deficiency led to a reduction in inflorescence dry mass of 49% (530 g), compared to well-fed plants (1020 g), and to disaggregated florets, reducing the marketability of the cauliflower heads (Fig 7). P and N improve the yield of cauliflower plants [20, 23] and are continually redistributed to the inflorescences, representing 30.0–40.5% of the total amount of P accumulated by cauliflower plants [13, 21, 22].

**Fig 8:**
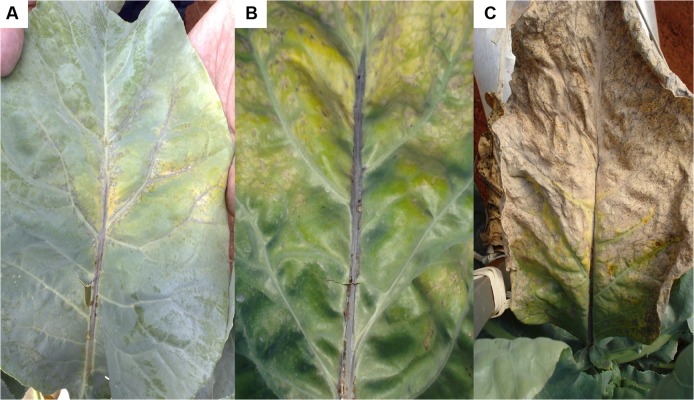
Progressive symptoms of K deficiency in the leaves of the cauliflower ‘Verona’ after being supplied with a nutrient solution without K. Chlorosis and initial necrosis of the nervures (A), advanced stage of necrosis of the nervures (B), and advanced foliar necrosis (C).

The Initial symptoms of K deficiency were observed 28 days after the plants were supplied with a nutrient solution without K. The plants were 77 days old and had 16 leaves and a leaf area of 68.39 dm^2^. Chlorosis of the boards of old leaves was initially observed, developing further to necrosis of the nervures and total foliar necrosis (Fig 8). Progressive necrosis occurred in the main and secondary nervures, and the leaves cracked. The symptoms of K deficiency were more delayed than those of N and P deficiency, because the period of higher demand for K per plant is later than for N and P. Takeishi et al. [13] observed a higher accumulation of K between 97 and 120 days after cauliflower sowing, while the accumulation of N and P was more than a week before that of K, corroborating the observations by Chatterje et al. [10]. Avalhães et al. [4], however, reported chlorosis of the boards of old leaves with greener central nervures. K deficiency leads to a lower synthesis of proteins and a higher accumulation of soluble nitrogenous compounds (putrescine, N-carbamoylputrescine, and agmatine); putrescine is toxic to plants and causes the necrosis of foliar tissues [18].

**Fig 9:**
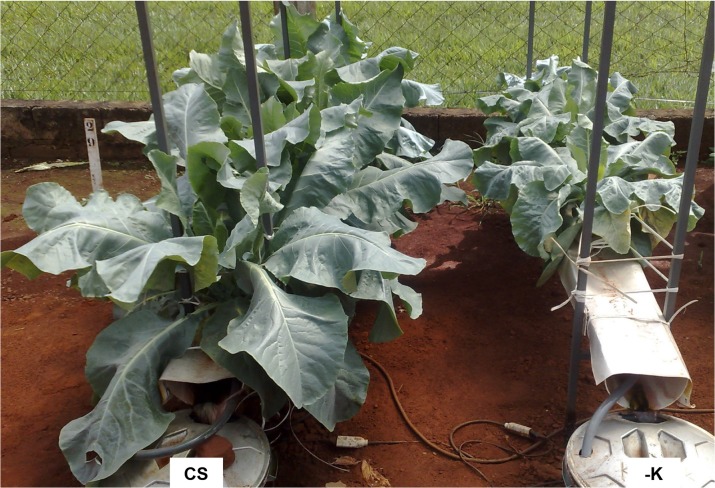
Plants of the cauliflower ‘Verona’ 45 days after being supplied with a complete nutrient solution (CS) and a nutrient solution without K (-K).

Twenty-eight days after the plants were supplied with a nutrient solution without K, when the initial deficiency symptoms were observed, the K contents of old, intermediate, and young leaves were 6.3, 6.5, and 8.6 g kg^-1^, respectively (Table 1 and S3 Table). The K content was 27.9 g kg^-1^ in a standard leaf of plants supplied with a complete nutrient solution, below an adequate range of 30–50 g kg^-1^ for well-fed plants [6]. Avalhães et al. [4] found 4.7 g kg^-1^ of K in the foliar tissues of K-deficient plants. Furlani et al. [9] and Avalhães et al. [4] reported K contents of 25.4 g kg^-1^ and 37 g kg^-1^, respectively, in the foliar tissues of cauliflower plants supplied with complete nutrient solutions. Old leaves have lower K contents than do intermediate and young leaves, likely due to a high mobility and redistribution of K within plants [15], and K-deficient plants have lower N, P, and Mg contents in old and intermediate leaves than do well-fed plants. As a consequence of K deficiency, cauliflower plant growth was lower than in well-fed plants 45 days after being supplied with a nutrient solution without K (Fig 9), corroborating the results of Avalhães et al. [4].

**Fig 10:**
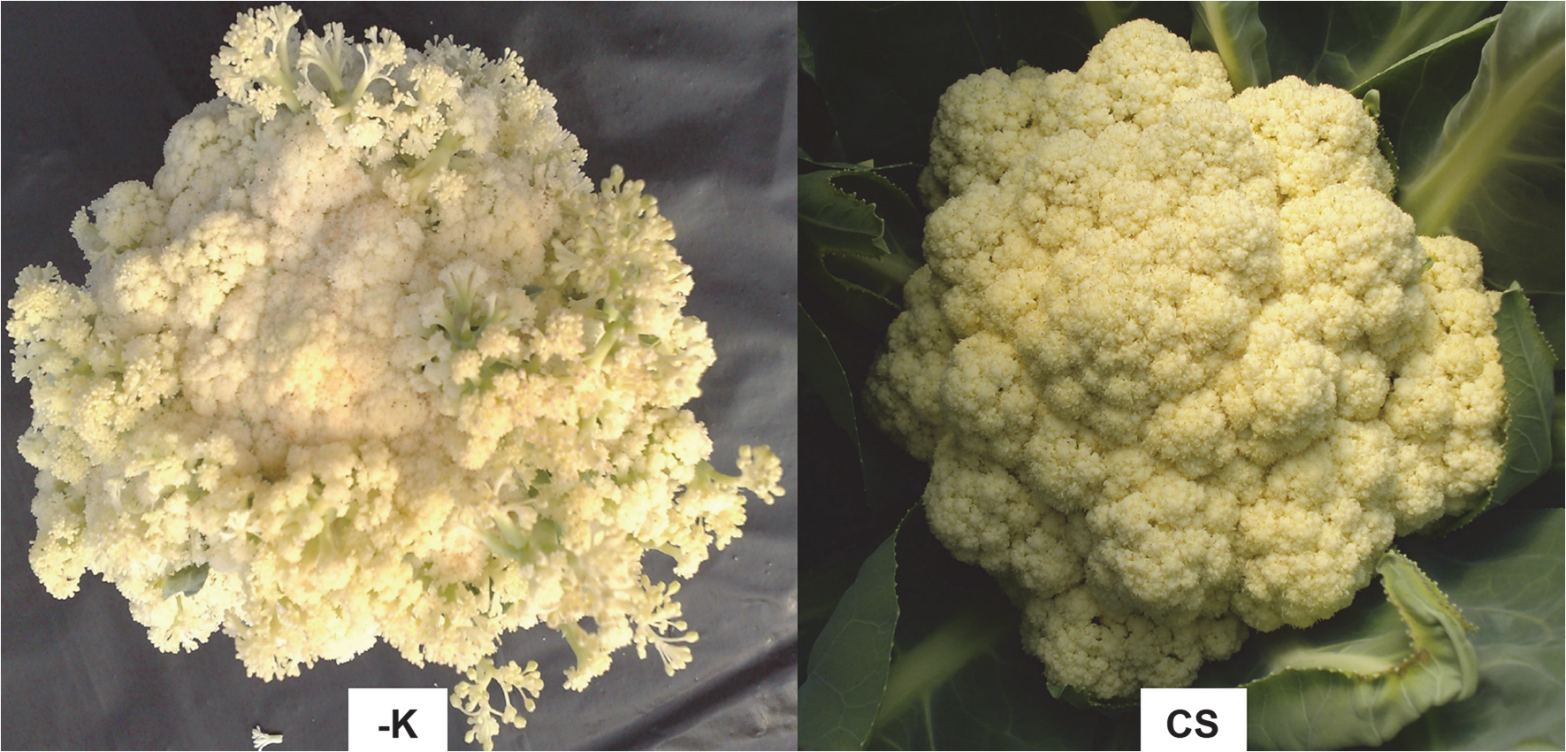
Detail of the cauliflower ‘Verona’ head at harvest supplied with a nutrient solution without K (-K) and a complete nutrient solution (CS).

At harvest, the K contents of old, intermediate, and young leaves were 1.8, 2.7, and 6.0 g kg^-1^, respectively (Table 1 and S3 Table). The K contents of inflorescences were also lower in plants grown without added K (21.6 g kg^-1^) than in plants supplied with a complete nutrient solution (38.2 g kg^-1^) (Table 2 and S4 Table). As a consequence of K deficiency, the cauliflower heads were not uniform (Fig 10), and inflorescence dry mass was reduced by 42% (580 g) compared to plants supplied with a complete nutrient solution (1020 g). The reduction in inflorescence dry mass was less than that observed in N- and P-deficient plants, corroborating the results of Cutcliffe et al. [23], likely due to the redistribution of K. Sánches et al. [21], Castoldi et al. [22], and Takeishi et al. [13] reported that 31.2, 30.0, and 55.2%, respectively, of the K at harvest was in the inflorescences.

**Fig 11:**
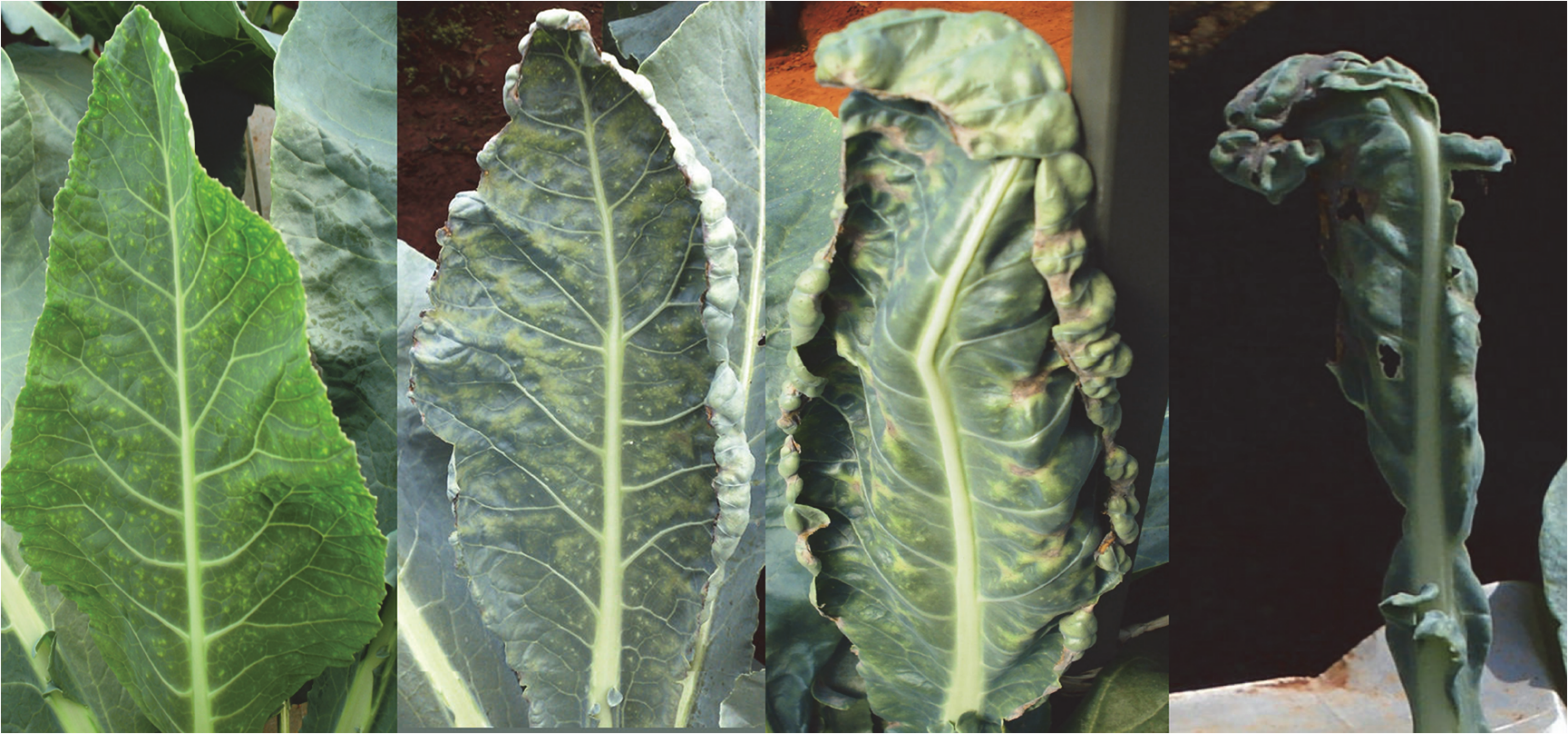
Progressive symptoms of Ca deficiency in the leaves of the cauliflower ‘Verona’, culminating in tip burn, supplied with a nutrient solution without Ca.

The initial symptoms of Ca deficiency were observed 40 days after the plants were supplied with a nutrient solution without Ca. The plants were 88 days old and had 17 leaves and a leaf area of 72.83 dm^2^. Chlorosis of the boards of young leaves was initially observed, followed by progressive chlorotic mottling and necrosis of the foliar tissues (Fig 11) and culminating in tip burn, a typical symptom of Ca deficiency [23, 30]. The Ca-deficient plants were smaller than well-fed plants by 10 days after the symptoms first appeared, due to the limitation of expansion of young leaves by the tip burn. Advanced Ca deficiency caused wrinkling in young leaves, but no symptoms were observed in intermediate and old leaves at harvest. Avalhães et al. [4] also observed a reduction in the number of leaves, plant height, stem diameter, aboveground dry mass, and root dry mass. Chaterjee et al. [10] reported the death and detachment of foliar tissues.

The chlorosis and necrosis in young leaves of Ca-deficient plants is due to the poor formation of cell walls, because Ca is essential for maintaining the integrity of membranes [12]. Ca also increases the tolerance of plants to abiotic stresses, such as salinity and cold weather [31–34], and to biotic stresses, such as parasitic diseases, due to its ability to strengthen cell walls and to prime defense against pathogen attack [30].

Forty days after the plants were supplied with a nutrient solution without Ca, when the initial deficiency symptoms were observed, the Ca contents of old, intermediate, and young leaves were 21.9, 7.8, and 5.4 g kg^-1^, respectively (Table 3 and S6 Table). The Ca content was 22.4 g kg^-1^ in a standard leaf of plants supplied with a complete nutrient solution, within an adequate range of 20–35 g kg^-1^ for well-fed-plants [6]. Avalhães et al. [4] found 5.6 g kg^-1^ of Ca in the leaves of Ca-deficient plants but 26.0 g kg^-1^ of Ca in the leaves of plants supplied with a complete nutrient solution. Old leaves were not deficient in Ca despite the lower Ca contents of intermediate and young leaves. Ca has very low, if any, mobility within plant tissues, so it is not redistributed from old to young leaves. The symptoms of Ca deficiency thus first appear in young leaves [12].

**Table 3:** Content of Ca and Mg in leaves of cauliflower supplied with omission of some macronutrient.

NS		Ca					Mg			
		OL	IL		YL		OL		IL	YL
**First Collection** ^**1**^										
**C**	22.4 c		22.4 a	22.4 a		6.0 b		6.0 a		6.0 ab
**-N**	4.3 d		6.3 b	4.3 d		1.6 c		1.2 c		2.8 c
**-P**	41.2 a		19.9 a	8.7 c		2.2 c		3.6 bc		6.9 a
**-K**	31.9 b		22.7 a	17.7 b		4.4 b		5.4 ab		7.2 a
**-Ca**	21.9 c		7.8 b	5.4 cd		10.5 a		6.0 a		5.6 b
**-Mg**	33.7 b		13.4 b	5.1 cd		0.8 c		1.4 c		4.7 b
**Second Collection** ^**2**^										
**C**	96.3 a		37.9 a	30.1 a		12.7 b		8.9 b		7.3 b
**-N**	13.4 d		7.4 c	5.8 c		2.1 cd		1.3 c		1.5 d
**-P**	21.9 cd		12.7 bc	7.4 bc		3.7 c		2.7 c		1.9 d
**-K**	40.4 b		27.9 a	19.0 ab		11.5 b		7.3 b		5.8 c
**-Ca**	20.5 cd		12.5 bc	6.0 c		23.0 a		19.0 a		12.0 a
**-Mg**	35.4 bc		24.2 ab	17.4 bc		0.9 d		0.6 c		0.8 d

Ca and Mg contents (g kg^-1^) of old (OL), intermediate (IL), and young (YL) leaves of the cauliflower ‘Verona’ supplied with a complete nutrient solution (CS) or a nutrient solution without added macronutrients (-N,-P,-K,-Ca, and-Mg).

^1^ The first collection was performed when deficiency symptoms first appeared, 40 and 22 days after being supplied with nutrient solutions without Ca or Mg, respectively.

^2^ The second collection was performed at inflorescence harvest. Means followed by the same letter in the same column did not differ significantly by Tukey’s test at 5% probability.

**Fig 12:**
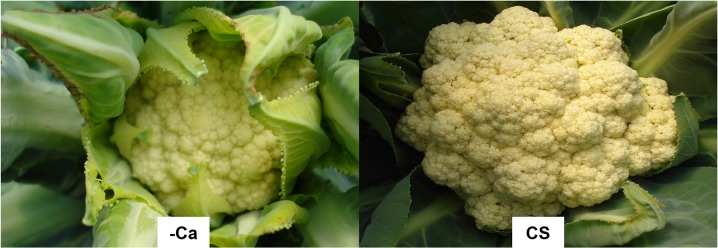
Detail of the cauliflower ‘Verona’ head at harvest supplied with a nutrient solution without Ca (-Ca), showing necrosis of the leaves covering the inflorescence, and supplied with a complete nutrient solution (CS).

The Ca contents of old, intermediate, and young leaves at harvest were 20.5, 12.5, and 6.0 g kg^-1^, respectively (Table 3 and S6 Table). The Ca contents of inflorescences were lower in plants grown without added Ca (1.1 g kg^-1^) than in plants supplied with a complete nutrient solution (4.8 g kg^-1^) (Table 2 and S5 Table). Ca deficiency reduced inflorescence dry mass by 20% (820 g) compared to plants supplied with a complete nutrient solution (1020 g). The reduction was not as severe as that caused by N, P, or K deficiencies, likely due to the low demand of Ca for inflorescence growth. Inflorescences of cauliflower plants contain only 1.1–6.5% of the Ca accumulated by plants [13, 21, 22]. The appearance of Ca-deficient cauliflower heads was not affected as much as for N, P, and K deficiency, but the boards of the leaves covering the inflorescences were necrotic (Fig 12).

**Fig 13:**
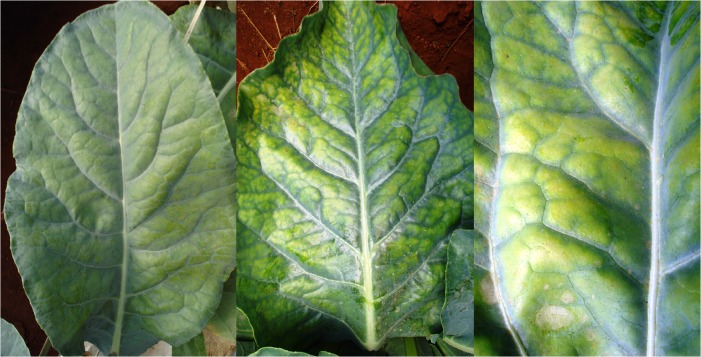
Progressive symptoms of Mg deficiency in the leaves of the cauliflower ‘Verona’ supplied with a nutrient solution without Ca.

The initial symptoms of Mg deficiency were observed 22 days after the plants were supplied with a nutrient solution without Mg. The plants were 70 days old and had 16 leaves and a leaf area of 72.83 dm^2^. Internerval chlorosis of old leaves was the first symptom to appear (Fig 13). A week later, the leaf blades became intensely yellow, the secondary nervures became light green, and the plants generally wilted. The chlorotic mottling became white, followed by the death of the foliar tissues. Avalhães et al. [4] reported that the omission of Mg caused wilting and chlorotic mottling between the nervures of old leaves, progressing to necrosis of the leaf boards. These symptoms were also observed by Chaterjee et al. [10], who also reported that leaf boards can become red and purple.

Foliar chlorosis occurs due to both a reduction in chlorophyll synthesis [29] and rapid chloroplast destruction. Many enzymes of the carboxylic acid cycle require Mg as an activator [12]. Chloroplasts in Mg-deficient leaves synthesize large starch grains; the grana become reduced, irregular, and vacuolated, so that chloroplast membranes are sometimes disrupted [10]. Mg also has a structural function in the chlorophyll molecule and an enzymatic function in phosphorylation reactions [15], which influence photosynthesis, respiration, and the synthesis of organic compounds, so that higher amounts of Mg is found within leaves. Leaves have the highest percentage of Mg in plant tissues, from 79.6–93.0% [13, 21, 22].

Twenty-two days after the plants were supplied with a nutrient solution without Mg, when the initial deficiency symptoms were observed, the Mg contents of old, intermediate, and young leaves were 0.8, 1.4, and, 4.7 g kg^-1^, respectively (Table 3 and S7 Table). The Mg content was 6 g kg^-1^ in a standard leaf of plants supplied with a complete nutrient solution, within an adequate range of 4–7 g kg^-1^ for well-fed plants [6]. Avalhães et al. [4] found 1.0 g kg^-1^ of Mg in Mg-deficient cauliflower plants, and Furlani et al. [9] suggested a critical Mg content of 1.7 g kg^-1^. Avalhães et al. [4], however, found 5.3 g kg^-1^ of Mg in the leaves of cauliflower plants supplied with a complete nutrient solution. Mg is highly mobile within plants, and the lower Mg contents of old and intermediate leaves of Mg-deficient plants are due to the redistribution of Mg to young leaves [12].

The Mg contents of old, intermediate, and young leaves at harvest were 0.9, 0.6, and 0.8 g kg^-1^, respectively (Table 3 and S7 Table). The Mg contents of the inflorescences were lower in plants without added Mg (0.7 g kg^-1^) than plants supplied with a complete nutrient solution (2.1 g kg^-1^) (Table 2 and S5 Table). Cauliflower is considered to be sensitive to Mg deficiency [35]. The accumulation of inflorescence dry mass was 20% lower in Mg-deficient plants (820 g) than in well-fed plants (1020 g), but the heads appeared similar to those of the well-fed plants. The small reduction in dry mass may be related to the redistribution of Mg to the inflorescence and to the small amount of Mg required for inflorescence development [13, 21, 22].

## Conclusions

Nitrogen deficiency caused chlorosis followed by purple color in the old leaves, while P deficiency caused only chlorosis in old leaves. Chlorosis at the edge of old leaves progressing to the center of the leaves was observed with the omission of K, and after was observed necrosis in the chlorotic areas. Ca deficiency caused tip burn in new leaves, while Mg deficiency caused internerval chlorosis in old leaves.

Foliar N, P, K, Ca, and Mg concentrations were 8.8, 0.5, 6.3, 5.4, and 0.8 g kg^-1^, respectively, in the corresponding treatments that did not supply these nutrients. The N, P, K, Ca, and Mg contents of raw dried inflorescences at harvest were 6.0, 0.5, 1.8, 6.0, and 0.9 g kg^-1^, respectively, in the corresponding treatments that did not supply these nutrients. The treatments without added N, P, and K most affected the growth and production of the plants, with inflorescence masses 87, 49, and 42%, respectively, lower than those of well-fed plants.

## Supporting Information

S1 Table Values observed of content (g kg^-1^) of N in older (OL), intermediate (IL), and younger (YL) leaves of cauliflower ‘Verona’ growing under supplying a complete nutrient solution (C) or a nutrient solution with omission of some macronutrient (-N,-P,-K,-Ca, and-Mg). (DOCX)

S2 Table Values observed of content (g kg^-1^) of P in older (OL), intermediate (IL), and younger (YL) leaves of cauliflower ‘Verona’ growing under supplying a complete nutrient solution (C) or a nutrient solution with omission of some macronutrient (-N,-P,-K,-Ca, and-Mg). (DOCX)

S3 Table Values observed of content (g kg^-1^) of K in older (OL), intermediate (IL), and younger (YL) leaves of cauliflower ‘Verona’ growing under supplying a complete nutrient solution (C) or a nutrient solution with omission of some macronutrient (-N,-P,-K,-Ca, and-Mg). (DOCX)

S4 Table Values observed of content of N, P, and K in inflorescence of cauliflower supplied with omission of some macronutrient. (DOCX)

S5 Table Values observed of content of Ca and Mg in inflorescence of cauliflower supplied with omission of some macronutrient. (DOCX)

S6 Table Values observed of content (g kg^-1^) of Ca in older (OL), intermediate (IL), and younger (YL) leaves of cauliflower ‘Verona’ growing under supplying a complete nutrient solution (C) or a nutrient solution with omission of some macronutrient (-N,-P,-K,-Ca, and-Mg). (DOCX)

S7 Table Values observed of content (g kg^-1^) of Mg in older (OL), intermediate (IL), and younger (YL) leaves of cauliflower ‘Verona’ growing under supplying a complete nutrient solution (C) or a nutrient solution with omission of some macronutrient (-N,-P,-K,-Ca, and-Mg). (DOCX)
