# Supplementary material for: Nutritional Status of the Cauliflower Cultivar ‘Verona’ Grown with Omission of out Added Macronutrients
Source: PLoS One. 2015 Apr 9;10(4):e0123500. doi: 10.1371/journal.pone.0123500 (PMC4391927; doi:10.1371/journal.pone.0123500)
Supplement: S1 Table — (DOCX) [file pone.0123500.s001.docx]

Table S1. Values observed of content (g kg^-1^) of N in older (OL), intermediate (IL), and younger (YL) leaves of cauliflower ‘Verona’ growing under supplying a complete nutrient solution (C) or a nutrient solution with omission of some macronutrient (-N, -P, -K, -Ca, and -Mg).

| **NS** | | | **OL** | | | | | **IL** | | | | **YL** | | | | | |
| --- | --- | --- | --- | --- | --- | --- | --- | --- | --- | --- | --- | --- | --- | --- | --- | --- | --- |
|  |  |  | **A** | | **B** | | **C** | **A** | **B** | | **C** | **A** | | | **B** | | **C** |
|  | | | **First Collection^1^** | | | | | | | | | | | | | | |
| **C** | | | 44,2 | | 44,2 | | 44,2 | 44,2 | 44,2 | | 44,2 | 44,2 | | | 44,2 | | 44,2 |
| **- N** | | | 4,9 | | 11,2 | | 10,2 | 12,3 | 13,3 | | 13,0 | 28,7 | | | 30,1 | | 24,2 |
| **- P** | | | 23,1 | | 21,4 | | 18,2 | 30,8 | 29,8 | | 23,1 | 31,9 | | | 31,9 | | 28,4 |
| **- K** | | | 28,0 | | 33,6 | | 27,3 | 35,0 | 34,0 | | 31,5 | 45,2 | | | 40,6 | | 40,6 |
| **- Ca** | | | 58,5 | | 60,2 | | 56,9 | 44,1 | 41,9 | | 46,3 | 37,1 | | | 39,4 | | 34,8 |
| **- Mg** | | | 23,5 | | 44,5 | | 21,0 | 24,5 | 36,4 | | 31,5 | 41,3 | | | 45,5 | | 45,5 |
|  | **Second Collection^2^** | | | | | | | | | | | | | | | |  |
| **C** | 25,2 | | 33,3 | | 28,0 | | 38,2 | 42,4 | 38,2 | | | 46,9 | 40,6 | | 2,5 | |  |
| **- N** | 6,7 | | 5,6 | | 5,6 | | 9,1 | 7,0 | 7,4 | | | 12,6 | 3,9 | | 11,2 | |  |
| **- P** | 32,2 | | 34,7 | | 34,7 | | 27,3 | 32,6 | 32,2 | | | 26,6 | 26,3 | | 28,4 | |  |
| **- K** | 26,6 | | 31,9 | | 27,7 | | 33,6 | 35,0 | 27,0 | | | 49,0 | 42,7 | | 45,9 | |  |
| **- Ca** | 28,4 | | 25,1 | | 31,8 | | 32,6 | 33,8 | 31,4 | | | 34,0 | 35,9 | | 31,9 | |  |
| **- Mg** | 32,6 | | 30,8 | | 36,4 | | 45,5 | 42,0 | 43,1 | | | 49,0 | 48,3 | | 46,2 | |  |

N contents (g kg^-1^) of old (OL), intermediate (IL), and young (YL) leaves of the cauliflower ‘Verona’ supplied with a complete(C) nutrient solution (NS) or a nutrient solution withoutadded macronutrients (-N, -P, -K, -Ca, and -Mg).

^1^ The first collection was performed when deficiency symptoms first appeared 15 days after being supplied with nutrient solutions without N.

^2^ The second collection was performed at inflorescence harvest.
