# Supplementary material for: Nutritional Status of the Cauliflower Cultivar ‘Verona’ Grown with Omission of out Added Macronutrients
Source: PLoS One. 2015 Apr 9;10(4):e0123500. doi: 10.1371/journal.pone.0123500 (PMC4391927; doi:10.1371/journal.pone.0123500)
Supplement: S2 Table — (DOCX) [file pone.0123500.s002.docx]

Table S2. Values observed of content (g kg^-1^) of P in older (OL), intermediate (IL), and younger (YL) leaves of cauliflower ‘Verona’ growing under supplying a complete nutrient solution (C) or a nutrient solution with omission of some macronutrient (-N, -P, -K, -Ca, and -Mg).

|  | | | **OL** | | | | | | **IL** | | | | **YL** | | | | | |
| --- | --- | --- | --- | --- | --- | --- | --- | --- | --- | --- | --- | --- | --- | --- | --- | --- | --- | --- |
|  |  |  | **A** | | **B** | | **C** | | **A** | **B** | | **C** | **A** | **B** | | **C** | | |
| **First Collection^1^** | | | | | | | | | | | | | | | | | | |
| **C** | | | 6,4 | | 6,4 | | 6,1 | | 6,4 | 6,4 | | 6,1 | 6,4 | 6,4 | | 6,1 | | |
| **- N** | | | 2,5 | | 3,1 | | 2,3 | | 2,7 | 3,1 | | 2,3 | 4,1 | 3,9 | | 3,2 | | |
| **- P** | | | 0,6 | | 0,4 | | 0,5 | | 0,6 | 0,4 | | 0,6 | 1,2 | 1,0 | | 1,0 | | |
| **- K** | | | 1,0 | | 5,2 | | 5,6 | | 1.0 | 5.6 | | 5.6 | 1,0 | 5,6 | | 5,6 | | |
| **- Ca** | | | 5.9 | | 5,5 | | 6,2 | | 5.6 | 5,3 | | 5,8 | 5.9 | 6,0 | | 5,8 | | |
| **- Mg** | | | 5,2 | | 4,1 | | 3,8 | | 5,2 | 5,0 | | 4,1 | 5,6 | 5,0 | | 5,0 | | |
| **Second Collection^2^** | | | | | | | | | | | | | | | | | |  |
| **C** | 6,1 | | 6,1 | | 6,1 | | 6,4 | | 5,9 | 6,1 | | 6,4 | | 6,4 | | 6,4 | |  |
| **- N** | 1,6 | | 1.4 | | 1,5 | | 2,0 | | 1,5 | 1,5 | | 2,6 | | 2,3 | | 2,0 | |  |
| **- P** | 0,4 | | 0,4 | | 0,6 | | 0,5 | | 0,4 | 0,5 | | 0,8 | | 0,6 | | 0,8 | |  |
| **- K** | 5,2 | | 6,4 | | 6,4 | | 4,7 | | 6,4 | 6,4 | | 5,4 | | 5,4 | | 5,6 | |  |
| **- Ca** | 5,4 | | 5,7 | | 5,2 | | 4,4 | | 4,0 | 4,8 | | 4,5 | | 4,2 | | 4,8 | |  |
| **- Mg** | 4,5 | | 3,9 | | 5,4 | | 4,5 | | 4,4 | 5,4 | | 6,1 | | 5,6 | | 6,1 | |  |

P contents (g kg^-1^) of old (OL), intermediate (IL), and young (YL) leaves of the cauliflower ‘Verona’ supplied with a complete(C) nutrient solution (NS) or a nutrient solution withoutadded macronutrients (-N, -P, -K, -Ca, and -Mg).

^1^ The first collection was performed when deficiency symptoms first appeared 19 days after being supplied with nutrient solutions without P.

^2^ The second collection was performed at inflorescence harvest.
