# Supplementary material for: Nutritional Status of the Cauliflower Cultivar ‘Verona’ Grown with Omission of out Added Macronutrients
Source: PLoS One. 2015 Apr 9;10(4):e0123500. doi: 10.1371/journal.pone.0123500 (PMC4391927; doi:10.1371/journal.pone.0123500)
Supplement: S3 Table — (DOCX) [file pone.0123500.s003.docx]

Table S3. Values observed of content (g kg^-1^) of K in older (OL), intermediate (IL), and younger (YL) leaves of cauliflower ‘Verona’ growing under supplying a complete nutrient solution (C) or a nutrient solution with omission of some macronutrient (-N, -P, -K, -Ca, and -Mg).

| **NS** | | | **OL** | | | | | | **IL** | | | | **YL** | | | | | |
| --- | --- | --- | --- | --- | --- | --- | --- | --- | --- | --- | --- | --- | --- | --- | --- | --- | --- | --- |
|  |  |  | **A** | | **B** | | **C** | | **A** | **B** | | **C** | **A** | | **B** | **C** | | |
| **First Collection^1^** | | | | | | | | | | | | | | | | | | |
| **C** | | | 31,5 | | 26,2 | | 25,9 | | 31,5 | 26,2 | | 25,9 | 31,5 | | 26,2 | 25,9 | | |
| **- N** | | | 12,0 | | 10,1 | | 9,5 | | 10,0 | 12,0 | | 10,4 | 21,4 | | 16,8 | 14,9 | | |
| **- P** | | | 22,9 | | 28,5 | | 18,8 | | 20,8 | 17,5 | | 15,0 | 18,9 | | 21,0 | 15,3 | | |
| **- K** | | | 17,2 | | 0,8 | | 1,0 | | 16,6 | 1,4 | | 1,5 | 14,6 | | 5,6 | 5,5 | | |
| **- Ca** | | | 31,6 | | 33,8 | | 29,5 | | 26,2 | 24,4 | | 28,1 | 33,5 | | 35,9 | 31,0 | | |
| **- Mg** | | | 20,2 | | 17,0 | | 16,1 | | 17,0 | 17,4 | | 14,8 | 19,6 | | 18,0 | 18,3 | | |
| **Second Collection^2^** | | | | | | | | | | | | | | | | | |  |
| **C** | 42,6 | | 24,9 | | 34,3 | | 35,6 | | 23,4 | 30,6 | | 32,7 | 29,9 | | | 26,6 | | |
| **- N** | 5,3 | | 6,7 | | 8,2 | | 11,0 | | 11,0 | 13,1 | | 19,2 | 17,8 | | | 17,8 | | |
| **- P** | 19,4 | | 23,3 | | 20,2 | | 17,5 | | 18,4 | 16,3 | | 18,5 | 19,7 | | | 19,4 | | |
| **- K** | 1,6 | | 1,9 | | 1,8 | | 2,7 | | 2,3 | 3,1 | | 8,9 | 5,3 | | | 3,9 | | |
| **- Ca** | 43,1 | | 40,1 | | 46,2 | | 33,5 | | 36,1 | 30,8 | | 25,4 | 28,1 | | | 22,6 | | |
| **- Mg** | 34,7 | | 22,7 | | 63,0 | | 36,4 | | 29,9 | 58,0 | | 33,9 | 29,1 | | | 38,4 | | |

K contents (g kg^-1^) of old (OL), intermediate (IL), and young (YL) leaves of the cauliflower ‘Verona’ supplied with a complete (C) nutrient solution (NS) or a nutrient solution withoutadded macronutrients (-N, -P, -K, -Ca, and -Mg).

^1^ The first collection was performed when deficiency symptoms first appeared 28 days after being supplied with nutrient solutions without K.

^2^ The second collection was performed at inflorescence harvest.
