# Supplementary material for: Nutritional Status of the Cauliflower Cultivar ‘Verona’ Grown with Omission of out Added Macronutrients
Source: PLoS One. 2015 Apr 9;10(4):e0123500. doi: 10.1371/journal.pone.0123500 (PMC4391927; doi:10.1371/journal.pone.0123500)
Supplement: S4 Table — (DOCX) [file pone.0123500.s004.docx]

Table S4. Values observed of content of N, P, and K in inflorescence of cauliflower supplied with omission of some macronutrient

| **NS** |  | **N** |  |  | **P** |  |  | **K** |  |
| --- | --- | --- | --- | --- | --- | --- | --- | --- | --- |
|  | **A** | **B** | **C** | **A** | **B** | **C** | **A** | **B** | **C** |
| **C** | 42,0 | 43,4 | 40,3 | 5,6 | 6,4 | 5,9 | 38,2 | 40,5 | 35,8 |
| **-N** | 27,3 | 29,0 | 25,6 | 5,0 | 5,9 | 4,1 | 32,9 | 32,1 | 33,7 |
| **-P** | 28,7 | 29,8 | 31,2 | 2,4 | 2,1 | 2,0 | 31,4 | 32,9 | 29,8 |
| **-K** | 70,0 | 72,8 | 72,5 | 6,4 | 6,3 | 6,5 | 23,4 | 19,7 | 21,8 |
| **-Ca** | 55,7 | 52,6 | 58,8 | 5,7 | 7,1 | 6,4 | 41,6 | 38,4 | 40,0 |
| **-Mg** | 52,9 | 39,6 | 42,0 | 6,4 | 6,6 | 6,2 | 29,8 | 37,9 | 40,0 |

N, P, and K contents (g kg^-1^) of inflorescences of the cauliflower ‘Verona’ supplied with a complete (C) nutrient solution (NS) or a nutrient solution without added macronutrients (-N, -P, -K, -Ca, and -Mg).
