# Supplementary material for: Nutritional Status of the Cauliflower Cultivar ‘Verona’ Grown with Omission of out Added Macronutrients
Source: PLoS One. 2015 Apr 9;10(4):e0123500. doi: 10.1371/journal.pone.0123500 (PMC4391927; doi:10.1371/journal.pone.0123500)
Supplement: S5 Table — (DOCX) [file pone.0123500.s005.docx]

Table S5. Values observed of content of Ca and Mg in inflorescence of cauliflower supplied with omission of some macronutrient

| **NS** |  | **Ca** |  |  | **Mg** |  |
| --- | --- | --- | --- | --- | --- | --- |
|  | **A** | **B** | **C** | **A** | **B** | **C** |
| **C** | 1,8 | 1,7 | 1,8 | 2,1 | 2,4 | 1,9 |
| **-N** | 3,2 | 2,8 | 3,0 | 2,2 | 1,8 | 2,5 |
| **-P** | 1,9 | 2,1 | 1,9 | 1,7 | 1,8 | 1,4 |
| **-K** | 2,8 | 3,9 | 5,4 | 2,3 | 2,4 | 2,9 |
| **-Ca** | 1,1 | 0,9 | 1,2 | 3,6 | 3,7 | 3,4 |
| **-Mg** | 6,6 | 4,0 | 3,9 | 0,7 | 0,7 | 0,6 |

Ca and Mg contents (g kg^-1^) of inflorescences of the cauliflower ‘Verona’ supplied with a complete (C) nutrient solution (NS) or a nutrient solution without added macronutrients (-N, -P, -K, -Ca, and -Mg).
