# Supplementary material for: Nutritional Status of the Cauliflower Cultivar ‘Verona’ Grown with Omission of out Added Macronutrients
Source: PLoS One. 2015 Apr 9;10(4):e0123500. doi: 10.1371/journal.pone.0123500 (PMC4391927; doi:10.1371/journal.pone.0123500)
Supplement: S6 Table — (DOCX) [file pone.0123500.s006.docx]

Table S6. Values observed of content (g kg^-1^) of Ca in older (OL), intermediate (IL), and younger (YL) leaves of cauliflower ‘Verona’ growing under supplying a complete nutrient solution (C) or a nutrient solution with omission of some macronutrient (-N, -P, -K, -Ca, and -Mg).

| **NS** | | | **OL** | | | | | | **IL** | | | | **YL** | | | | | |
| --- | --- | --- | --- | --- | --- | --- | --- | --- | --- | --- | --- | --- | --- | --- | --- | --- | --- | --- |
|  |  |  | **A** | | **B** | | **C** | | **A** | **B** | | **C** | **A** | **B** | | **C** | | |
| **First Collection^1^** | | | | | | | | | | | | | | | | | | |
| **C** | | | 26,2 | | 19,1 | | 21,8 | | 26,2 | 19,1 | | 21,8 | 26,2 | 19,1 | | 21,8 | | |
| **- N** | | | 4,8 | | 4,4 | | 3,7 | | 6,4 | 7,1 | | 5,4 | 17,7 | 17,4 | | 16,6 | | |
| **- P** | | | 9,7 | | 8,0 | | 8,3 | | 24,1 | 17,4 | | 18,3 | 36,3 | 48,1 | | 39,1 | | |
| **- K** | | | 8,0 | | 22,8 | | 22,3 | | 8,7 | 29,8 | | 29,7 | 9,6 | 46,2 | | 39,9 | | |
| **- Ca** | | | 21,9 | | 20,1 | | 23,8 | | 7,8 | 6,5 | | 9,2 | 5,4 | 4,3 | | 6,4 | | |
| **- Mg** | | | 8,0 | | 4,5 | | 2,8 | | 23,2 | 9,2 | | 7,8 | 48,2 | 27,8 | | 25,1 | | |
| **Second Collection^2^** | | | | | | | | | | | | | | | | | |  |
| **C** | 102,0 | | 95,0 | | 95,0 | | 42,2 | | 29,5 | 42,0 | | 31,6 | | 35,4 | | 23,3 | |  |
| **- N** | 16,4 | | 10,2 | | 13,5 | | 8,2 | | 6,4 | 7,5 | | 6,7 | | 5,1 | | 5,5 | |  |
| **- P** | 20,1 | | 22,0 | | 23,8 | | 10,8 | | 12,9 | 14,3 | | 6,4 | | 6,9 | | 8,8 | |  |
| **- K** | 38,4 | | 40,1 | | 42,8 | | 23,7 | | 25,9 | 34,3 | | 12,7 | | 20,8 | | 23,6 | |  |
| **- Ca** | 20,5 | | 23,4 | | 17,6 | | 12,5 | | 9,6 | 15,3 | | 6,0 | | 5,6 | | 6,5 | |  |
| **- Mg** | 44,1 | | 19,2 | | 42,9 | | 31,3 | | 14,0 | 27,4 | | 21,9 | | 9,7 | | 20,6 | |  |

Ca contents (g kg^-1^) of old (OL), intermediate (IL), and young (YL) leaves of the cauliflower ‘Verona’ supplied with a complete (C) nutrient solution (NS) or a nutrient solution without added macronutrients (-N, -P, -K, -Ca, and -Mg).

^1^ The first collection was performed when deficiency symptoms first appeared 40 days after being supplied with nutrient solutions without Ca.

^2^ The second collection was performed at inflorescence harvest.
