# Supplementary material for: Nutritional Status of the Cauliflower Cultivar ‘Verona’ Grown with Omission of out Added Macronutrients
Source: PLoS One. 2015 Apr 9;10(4):e0123500. doi: 10.1371/journal.pone.0123500 (PMC4391927; doi:10.1371/journal.pone.0123500)
Supplement: S7 Table — (DOCX) [file pone.0123500.s007.docx]

Table S7. Values observed of content (g kg^-1^) of Mg in older (OL), intermediate (IL), and younger (YL) leaves of cauliflower ‘Verona’ growing under supplying a complete nutrient solution (C) or a nutrient solution with omission of some macronutrient (-N, -P, -K, -Ca, and -Mg).

| **NS** | | **OL** | | | | | | **IL** | | | | **YL** | | | | | |
| --- | --- | --- | --- | --- | --- | --- | --- | --- | --- | --- | --- | --- | --- | --- | --- | --- | --- |
|  |  | **A** | | **B** | | **C** | | **A** | **B** | | **C** | **A** | **B** | | **C** | | |
| **First Collection^1^** | | | | | | | | | | | | | | | | | |
| **C** | | 6,7 | | 5,9 | | 5,5 | | 6,7 | 5,9 | | 5,5 | 6,7 | 5,9 | | 5,5 | | |
| **- N** | | 1,8 | | 1,6 | | 1,3 | | 1,3 | 1,2 | | 1,1 | 2,8 | 2,8 | | 2,9 | | |
| **- P** | | 2,4 | | 2,2 | | 1,9 | | 3,9 | 3,5 | | 3,4 | 6,1 | 8,0 | | 6,8 | | |
| **- K** | | 2,2 | | 5,4 | | 5,7 | | 2,0 | 7,2 | | 6,9 | 2,2 | 10,2 | | 9,3 | | |
| **- Ca** | | 10,5 | | 9,2 | | 11,8 | | 6,0 | 7,8 | | 4,3 | 5,6 | 4,7 | | 6,5 | | |
| **- Mg** | | 0,8 | | 0,8 | | 0,8 | | 2,8 | 0,7 | | 0,6 | 7,2 | 3,4 | | 3,5 | | |
| **Second Collection^2^** | | | | | | | | | | | | | | | | |  |
| **C** | 13,0 | 12,0 | | 6,6 | | 9,1 | | 7,1 | 10,4 | | 7,1 | | 8,2 | | 6,6 | |  |
| **- N** | 2,4 | 1,7 | | 2,1 | | 1,3 | | 1,2 | 1,4 | | 1,4 | | 1,6 | | 1,6 | |  |
| **- P** | 3,5 | 3,8 | | 3,9 | | 2,3 | | 2,9 | 2,9 | | 1,8 | | 1,8 | | 2,1 | |  |
| **- K** | 10,8 | 10,6 | | 13,0 | | 6,9 | | 6,5 | 8,5 | | 5,0 | | 5,8 | | 6,7 | |  |
| **- Ca** | 23,0 | 24,5 | | 21,6 | | 19,0 | | 20,9 | 17,1 | | 12,0 | | 10,7 | | 13,2 | |  |
| **- Mg** | 1,1 | 0,5 | | 1,1 | | 0,7 | | 0,5 | 0,7 | | 0,9 | | 0,8 | | 0,8 | |  |

Mg contents (g kg^-1^) of old (OL), intermediate (IL), and young (YL) leaves of the cauliflower ‘Verona’ supplied with a complete (C) nutrient solution (NS) or a nutrient solution without added macronutrients (-N, -P, -K, -Ca, and -Mg).

^1^ The first collection was performed when deficiency symptoms first appeared 22 days after being supplied with nutrient solutions without Mg.

^2^ The second collection was performed at inflorescence harvest.
